# Volatilomes of Bacterial Infections in Humans

**DOI:** 10.3389/fnins.2020.00257

**Published:** 2020-03-25

**Authors:** Moamen M. Elmassry, Birgit Piechulla

**Affiliations:** ^1^Department of Biological Sciences, Texas Tech University, Lubbock, TX, United States; ^2^Institute for Biological Sciences, University of Rostock, Rostock, Germany

**Keywords:** microbial volatiles, volatile organic compounds, human microbiome, MVOC, volatilome

## Abstract

Sense of smell in humans has the capacity to detect certain volatiles from bacterial infections. Our olfactory senses were used in ancient medicine to diagnose diseases in patients. As humans are considered holobionts, each person’s unique odor consists of volatile organic compounds (VOCs, volatilome) produced not only by the humans themselves but also by their beneficial and pathogenic micro-habitants. In the past decade it has been well documented that microorganisms (fungi and bacteria) are able to emit a broad range of olfactory active VOCs [summarized in the mVOC database (http://bioinformatics.charite.de/mvoc/)]. During microbial infection, the equilibrium between the human and its microbiome is altered, followed by a change in the volatilome. For several decades, physicians have been trying to utilize these changes in smell composition to develop fast and efficient diagnostic tools, particularly because volatiles detection is non-invasive and non-destructive, which would be a breakthrough in many therapies. Within this review, we discuss bacterial infections including gastrointestinal, respiratory or lung, and blood infections, focusing on the pathogens and their known corresponding volatile biomarkers. Furthermore, we cover the potential role of the human microbiota and their volatilome in certain diseases such as neurodegenerative diseases. We also report on discrete mVOCs that affect humans.

## Humans Recognize and Are Constantly Affected by Microbial Volatiles

Olfaction is one of the most prominent and specific sensory system in invertebrates and vertebrates. It is composed of species-specific olfactory organs and the central nervous system. To optimize their behaviors, animals and humans constantly survey and recognize chemical cues in their environment. Many of these stimuli are volatile organic compounds (VOCs), which are indispensable for the characterization of the surroundings and help to achieve certain functions (e.g., differentiate human kin from “others,” detect predators, or discriminate edible food from poisonous or spoiled food). It is well appreciated that VOCs originate from animals, humans, or plants, but only few smells were commonly acknowledged to be emitted from microbes. However, it is now well established that microbes emit a plethora of VOCs. This has been documented by an increasing number of entries in the microbial volatile organic compounds (mVOC) database (Lemfack et al., 2017). For example, the characteristic earthy smell of a forest is due to the mVOC, geosmin, which is released by bacterial species such as *Streptomyces* (reviewed in Piechulla and Lemfack, 2016). Other well-known examples include the fine aromas of wine or cheese, which are produced by microorganisms during the fermentation process. mVOC production serves similar informative and defensive functions as VOCs from eukaryotes, e.g., bacteria and fungi emit pungent smells that attract flies or prevent animal feeding on decaying food.

**FIGURE 1:**
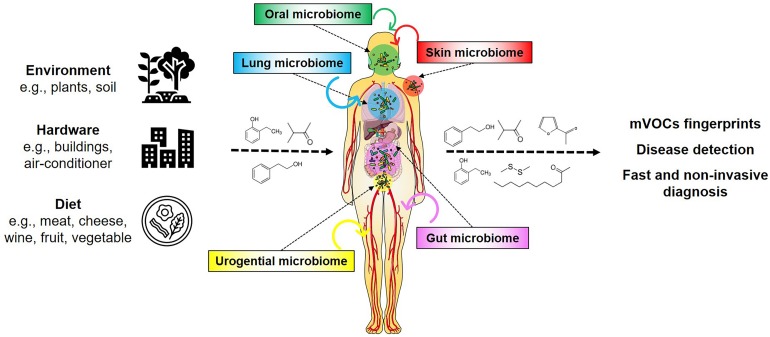
mVOCs and the human holobiont. Microorganisms appear universal in the environment, on hardware, on foodstuff and in/on humans. Their metabolism produces many compounds, including volatiles. These volatiles influence and affect humans. mVOCs released of the human microbiomes are potential biomarkers for non-invasive diagnosis.

The chemical diversity of mVOCs results from the substantial number and biodiversity of microbes living on Earth. Presently 10^6^ bacterial species are known while 10^16^ are estimated to exist on Earth (Pedrós-Alió and Manrubia, 2016; Larsen et al., 2017). So far (only) six out of 26 known bacterial phyla have been investigated regarding their volatile emission, corresponding to a very small proportion of known species (i.e., 0.00000006%) that is presently found in the database (Lemfack et al., 2020). It can be envisioned that the large number of diverse microbial genomes yet to be analyzed harbor hidden metabolic and physiological potentials, which could produce a large number of compounds during both primary and secondary metabolism, and under various nutritional conditions (Donia and Fischbach, 2015; Piechulla et al., 2017, 2020; Scott and Piel, 2019; Lemfack et al., 2020). These exceptional microbial features have become increasingly important with the realization that humans have to be considered as holobionts, as they contain communities of microorganisms within their bodies as well as on their skin (Grice et al., 2009; Grice and Segre, 2011; Marchesi, 2011; Bouslimani et al., 2015; Figure 1). It is assumed that these human-associated microorganisms produce low and high (the latter are not considered in this article) molecular weight compounds where they reside, but their influences on humans are not yet sufficiently studied.

### Biosynthesis of mVOCs

The microbial organic volatiles of bacteria are considered either as primary or as secondary metabolites, depending on whether they are produced during the exponential growth phase or during the transition to or in the stationary growth phase, respectively. The ambition of bacteria during primary metabolism is to gain as much ATP as possible. The level of generated ATP very much depends on the availability of electron acceptors. If oxygen or nitrate as final electron acceptor are available, glucose can be completely metabolized to the inorganic volatile CO_2_, while under oxygen limitation fermentation processes are activated, which finally lead to the production of acetate, ethanol, or formate. Beside the main alternative electron acceptors nitrate and sulfate, several other compounds can serve as electron sink during growth of certain bacteria. For example, dimethyl sulfoxide (DMSO) is reduced to dimethyl sulfide (DMS) and trimethyl amine-N-oxide (TMAO) to trimethyl amine (summarized in Lemfack et al., 2020). Sulfur compounds are common microbial volatiles, e.g., methanethiol, dimethyl disulfide (DMDS), dimethyl trisulfide (DMTS); the latter two compounds are derived from methanethiol.

The main carbon source for metabolic reactions is glucose, but aliphatic amino acids such as alanine, valine, leucine, and isoleucine are also preferred metabolites, which after transamination lead to keto acids that release CO_2_ to yield aldehydes (Schulz et al., 2020). These aldehydes can be oxidized to form either 2- and 3-methylbutanoic or isobutyric acids. Reductions deliver the respective alcohols. Alcohols and acids can be fused into esters usually by acyltransferases, leading to short chain esters. Terminal oxidation of acids followed by ring closure, results in lactones. Reactions of amines with aldehydes or acids lead to imines and amines. A carbon chain elongation results in elongated short chain branched acids (Schulz et al., 2020).

Fatty acid biosynthesis produces a wide variety of compounds of different chain length, of number and position of methyl branches and the oxidative status of functional groups. Fatty acids are produced from acetyl-CoA (in bacteria often also methyl butyryl-, isobutyryl-, or propanyl-CoA) by the typical polyketide extension reactions of the fatty acid synthase complex. Numerous derivatives may occur due to intermediate decarboxylation, reduction of methyl ketones, hydrolysis, and reduction reactions (see more details in Schulz et al., 2020). Polyketides are biosynthetically related to fatty acids, as they are synthesized by polyketide synthase complex (PKS). So far only very few volatiles of this compound class are known, one example is streptopyridine from *Streptomyces* sp. FORM5.

Alcohols, aldehydes, acids and esters and their derivatives are the dominant compound classes present in the mVOC database (Lemfack et al., 2017).

Aromatic compounds originate from the shikimate pathway. A very widespread volatile of this class is 2-phenylethanol formed from the amino acid phenylalanine or phenylpyruvate. Another well-known compound of this class is indole, characteristically produced by *Escherichia coli*. Derivatives of phenylpyruvate metabolism are methyl benzoate and acetophenone. Schleiferons are released from *Staphylococcus schleiferi* and biosynthesized by 2-phenylethylamine and acetoin (Lemfack et al., 2016; Kai et al., 2018).

Pyrazines are nitrogen containing aromatic compounds that are released by many bacteria. Pyrazines are often decorated with one to four methyl or ethyl groups, and are formed by condensation of acetoin, diketones, aminoaldehydes, or aminoketones. Since the latter intermediates are toxic, they are converted into non-toxic compounds.

Terpenes are well-known natural compounds amounting up to 50,000 compounds built from the C_5_ building blocks isopentenyl pyrophosphate (IPP) and dimethyl allyl pyrophosphate (DMAPP) (Christiansen, 2017). While the biosynthesis of plant terpenes by terpene synthases is quite well characterized, only ca. 60 terpene synthases are presently known from bacteria (primarily from Actinomycetes). The most prominent bacterial terpenes are geosmin and methyl isoborneol (summarized in Dickschat, 2016). Recently a sesquiterpene with a unique structure was isolated from *Serratia plymuthica* (von Reuss et al., 2010, 2018). Terpene synthases are fascinating enzymes as they are quite limited regarding their substrate usage (GPP, FPP, GGPP, NPP, and/or respective enantiomers), however their reaction mechanisms (including several cyclization cascades) can release a multitude of products (multi product enzymes). It is interesting to note, that the elucidation of the sodorifen biosynthesis led to a change of paradigm, as the substrate for the sodorifen terpene synthase is a methylated and cyclic FPP, produced by an upstream acting SAM-dependent *C*-methyltransferase (von Reuss et al., 2018).

As lined out above, the principles of many pathways of mVOC biosynthesis are understood, however the detailed elucidation of specific pathways or reaction steps may hide surprises, e.g., new reaction mechanisms and new catalytic enzymes.

The question why these volatile compounds are produced by the bacteria has to be answered case by case. One possibility is the incomplete (primary) metabolism occurring under certain metabolic conditions, e.g., depletion of carbon sources, lack of electron acceptors, lack of enzyme repertoire. Alternatively, the biosynthesis of mVOCs can be induced and produced due to environmental cues and/or presence of co-inhabitants of an ecological niche to defend or attract kins, conspecifics, or neighbors. Elucidation of the interactions of co-inhabitants will shine light on the ecological networks and lead to the understanding of the metabolic reactions of the players within a habitat. Alternatively, clarifying the regulation of the biosynthesis of mVOCs will also contribute to this general understanding. Presently, not much work has been performed in this regard, but principal regulatory mechanisms such as quorum sensing and catabolite repression turn out to be involved, as shown for the biosynthesis of schleiferon (Lemfack et al., 2016) and sodorifen (Magnus et al., 2017), just to name two examples.

### mVOCs of the Environment Affect Humans

mVOCs are products of microbial metabolism. Since microorganisms appear universal, no natural habitat can be considered sterile. Even extreme locations (hot springs, sites with extremely low pH or at low temperatures, e.g., glaciers and icebergs) are populated with more or less complex microbial communities. Furthermore, microbes often cover hardware using their ability to form biofilms (e.g., air conditioners, heat exchanger fins of evaporators from cars, medical tubing). An exception is pure metal surfaces (coins, products made from steel, etc.) or objects that were sterilized and kept sterile. Very pronounced sites of microbial biofilms are bacteria and fungi growing on moist indoor walls, ceilings, or furniture. These microbes release many well-known mVOCs that affect humans and are considered hazardous to health. Primarily eyes and the upper respiratory tract are attacked, supporting the term “sick building syndrome” that was coined in the last decade and refers to a set of symptoms that are experienced by residents living in rooms with low air quality (Polizzi et al., 2012). Despite the lack of studies on concentration-response relationships, mVOCs have been associated with general discomfort i.e., headache, dizziness, and fatigue. Korpi et al. (2009) evaluated 15 indoor mVOCs in detail. Although from the experimental exposure studies it turned out that symptoms of irritation appeared at mVOC concentrations several orders of magnitude higher than those measured indoors, “sick building syndrome” likely results from a combination of factors: (1) occupants are exposed to low quality air for very long periods, (2) the mVOCs may come from various sources, such as building materials, human activities, traffic, foodstuff, smoking and may overlap and act additively/synergistically in mixtures, and (3) taking into account, that the hazardous compounds may occur at low levels and were so far not detectable with the present technical approaches. It furthermore has to be considered that the main mVOCs produced by biofilms of building materials (3-methyl-1-butanol, 1-pentanol, and 1-octen-3-ol) are not reliable indicators for biocontamination (Korpi et al., 1998), although Inamdar et al. (2010, 2013) tested low vapor concentrations of C_8_-compounds, including 1-octen-3-ol, and showed toxicity to *Drosophila melanogaster* flies and larvae. A major goal for the future is to determine reliable markers for indoor-air environment relevant for humans. It will be important to detect hidden microbial growth as early as possible to improve indoor air quality immediately.

Due the presence of microorganisms, foodstuff such as vegetables, fruits, meat, dairy products, and cheese have a limited storage life. Carbohydrates, proteins, and fatty acids within the food are excellent nutrient sources for microbial growth. The microbial metabolism converts these compounds into microbial biomass and some reaction products are released as metabolites, including the mVOCs. Some of these mVOCs can be detected by the human nose, but there is a given detection limitation regardless of whether the mVOCs have a pleasant or pungent smell.

A valuable aroma is released by truffle species. They emit up to 200 VOCs many of them have a chain length of eight carbon atoms (i.e., characteristic mushroom VOCs). The distinct species differ in their scent spectra, e.g., *Tuber borchii* and *Tuber melanosporum* are distinct to *Tuber indicum* and *Tuber magnatum* (Splivallo et al., 2007). Furthermore, it was shown that truffles host various yeasts and bacteria (Vahdatzadeh et al., 2015) and e.g., the cyclic sulfur volatiles (thiophene derivatives) derive from bacteria living in/on the white truffle *T. borchii* (Splivallo et al., 2014; Splivallo and Ebeler, 2015). While remarkably interesting for the food industry, the biological and ecological functions of the majority of the mVOCs are still unknown, although dimethyl sulfide (DMS) was convincingly shown to attract mammals (Talou et al., 1990).

Odorous compounds from wine, primarily esters such as acetic ester, ethyl ester and VOCs such as ethyl hexanoate and ethyl octanoate are the dominant compounds recognized by humans. Defined bacterial starter cultures are applied to grape juices and the fermentation processes are controlled to reveal specific wine aromas.

More than 100 volatiles are present in yogurt and other dairy products, which are produced from lipolysis of milkfat and microbial transformation of lactose and citrate (Cheng, 2010). Particularly acetaldehyde, diacetyl, acetoin, acetone, and butanone are important odoriferous volatiles, while off-flavor compounds due to lipid oxidation appear during extended shelf-lives. Typical milk fermenting bacteria are *Leuconostoc lactis*, *Lactobacillus* sp., *Brachybacterium* sp., *Brevibacterium* sp., and *Propionibacterium* sp., each emitting a specific spectrum of VOCs.

Rotting fruits and vegetables, and spoiled meat and fish also release typical off-flavor VOCs, some of them can be recognized by humans as typical markers for decaying food. For example, the European sea bass is a popular farmed fish due to its white flesh and low fat, but microorganisms grow on the fish and produce characteristic off-odors (e.g., trimethylamine, dimethylamine, and ammonia) resulting in organoleptic rejection (Parlapani et al., 2015). Other mVOCs have been reported as metabolites released during meat spoilage by *Pseudomonas* sp., *Brochothrix thermosophacta*, *Schewanella* sp., and Enterobacteriaceae. Fruit sugars are fermented by fungi and bacteria into long-chain fatty acids and respective esters, which attract flies and other invertebrates but are pungent smells to the human nose.

*Streptomyces* sp., *Anabaena* sp., and *Oscillatoria* sp. are typical soil or aquatic inhabitants that produce the well-known off-flavors, geosmin or 2-methyl isoborneol, which are recognized by humans as muddy and earthy smells. Drinking water with this earthy smell is immediately identified as being “contaminated by bacteria.”

### mVOCs of Human Microbiomes Affect Humans

Scents of plants are widely used in well-being aroma therapies, but the medical potential of mVOCs is less explored, due to the historical observation that single or repeated exposure to mVOCs lead to negative influences, such as irritations, narcosis, central nervous system disturbances, or death (Andrew and Snyder, 1991). In general humans complain with eye, nose, throat, or asthma-like symptoms when exposed to mVOCs, however, there is a lack of studies on dose-effects or dose-responses regarding irritations from single or mixtures of mVOCs (Korpi et al., 2009). Furthermore, studies of mVOCs with humans under different conditions and experimental set ups reduce comparability. Moreover, in many animal and *in vitro* studies, LD_50_ (lethal doses) of single or multiple administrations, or single or multiple inhalations were determined (in Korpi et al., 2009). 3-Methyl-2-butanone and 3-methyl-2-butanol were tested positive in the mutagenicity tests (Ames test), while SOS-inducing activity due to DNA damage was reported for 2-methyl-1-propanol, 3-methyl-1-butanol, 3-methyl-2-butanol, 2-pentanol, 3-octanol, 1-octen-3-ol, 2-hexanone, 2-heptanone, 3-octanone only under cytotoxic conditions. It was verified that effects at higher exposure concentrations might even show synergistic interactions (Korpi et al., 1999). It was recently shown for the first time that skin-resident Corynebacterial and Staphylococcal species emit volatiles, including the new compounds schleiferon A and B [3-(phenylamino/imino)butan-2-one, respectively]. The latter compounds are emitted by *Staphylococcus schleiferi* and have the potential to affect quorum sensing- dependent phenotypes, prodigiosin accumulation, and bioluminescence of Gram-negative bacteria, while inhibiting the growth of Gram-positive species, ultimately modulating differentially and specifically the members of a bacterial (skin) community (Lemfack et al., 2016). In future, human cell lines might be promising test systems, since e.g., propionic acid, valeric and isovaleric acid, butyric and isobutyric acid, and acetic acid, released from *Porphyromonas* sp., *Prevotella* sp., and *Fusobacterium* sp., inhibit lymphocyte proliferation and cytokine production (Kurita-Ochiai et al., 1995).

## Pathogenic Bacteria and Their Unique Infection Volatilomes in Humans

**FIGURE 2:**
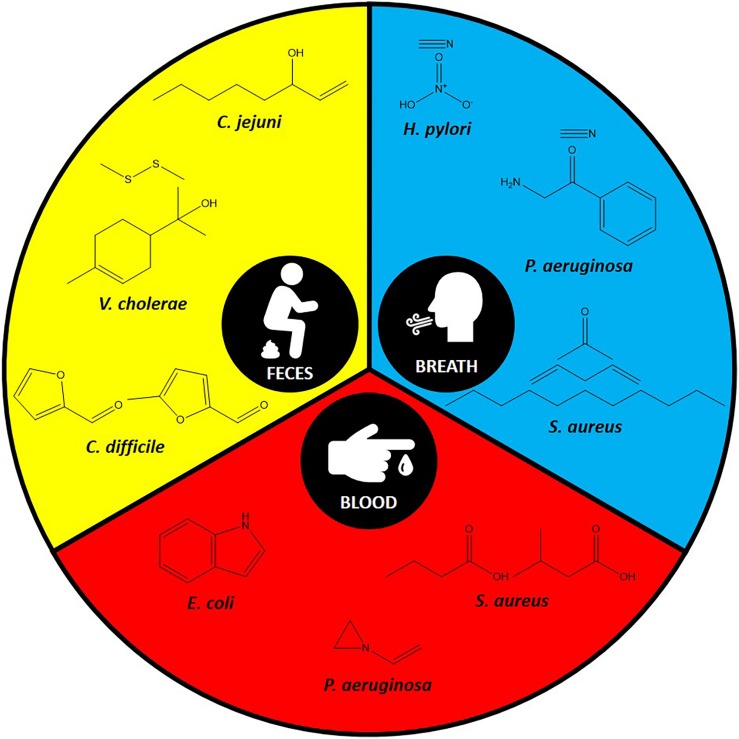
Some of the specific volatiles identified in the most commonly used specimens for each pathogen.

With the advances in volatiles detection technologies, there is increasing interest in linking bacteria and their associated unique volatiles in the context of human infection for better diagnosis. Environmental factors and nutrient availability are critical stimuli for the production of unique volatiles in bacteria. Therefore, here we focus on the unique volatiles produced by certain bacterial species during human infections. These include gastrointestinal, respiratory, lung, and bloodstream infections, as summarized in Table 1 and Figure 2. Although the hope is to use these volatiles in diagnostics, it is important to consider the original role of these volatiles, which could be for signaling or virulence.

**TABLE 1:** A summary of identified volatiles for each pathogen and type of infection.

Pathogen	Infection	Volatiles	Specimen	References
*Clostridioides difficile*	Gastrointestinal	2-furancarboxaldehyde; 5-methyl-2-furancarboxaldehyde	Feces	Probert et al., 2004
		propan-1-ol; 3-methylbutanal; ethyl propionate; hexanoic acid; *p*-cresol; dodecane; indole		Patel et al., 2019
*Vibrio cholerae*		dimethyl disulfide; *p*-menth-1-en-8-ol		Garner et al., 2009
*Campylobacter jejuni*		1-octen-3-ol		Garner et al., 2007
*Helicobacter pylori*		hydrogen nitrate; hydrogen cyanide	Breath	Lechner et al., 2005
*Staphylococcus aureus*	Respiratory	undecane; 1,4-pentadiene; acetone		Neerincx et al., 2016
*Pseudomonas aeruginosa*		methyl thiocyanate		Shestivska et al., 2011
		hydrogen cyanide		Gilchrist et al., 2013; Smith et al., 2013
		2-aminoacetophenone		Scott-Thomas et al., 2010
		2-hexanone	Sputum	Goeminne et al., 2012
		2-nonanone		Savelev et al., 2011
		2-butanone; 3-methyl-2-butanone	Bronchoalveolar lavage	Nasir et al., 2018
*Acinetobacter baumannii*		1-undecene; nonanal; decanal; 2,6,10-trimethyl-dodecane; 5-methyl-5-propyl-nonane; longifolene; tetradecane; 2-butyl-1-octanol	Breath	Gao et al., 2016
*Mycobacterium tuberculosis*		naphthalene; 1-methyl-cyclohexane; 1,4-dimethyl-cyclohexane		Phillips et al., 2007
		methyl phenyl-acetate; methyl nicotinate; methyl p-anisate; o-phenylanisole		Syhre and Chambers, 2008
*Escherichia coli*	Bloodstream	dimethyl sulfide; carbon disulfide; ethanol; acetaldehyde; methyl butanoate	Blood	Umber et al., 2013
		indole		Zhong et al., 2019; Chingin et al., 2015
		acetaldehyde; ethanol; acetone; hydrogen sulfide; methanethiol; dimethyl sulfide		Allardyce et al., 2006
*Pseudomonas aeruginosa*		acetic acid; acetone		Allardyce et al., 2006
		1-vinyl aziridine; trimethylamine		Chingin et al., 2015
*Staphylococcus aureus*		butyric acid; isovaleric acid		Chingin et al., 2015
		acetaldehyde; ethanol; ammonia; methanethiol; dimethyl sulfide		Allardyce et al., 2006
*Acinetobacter baumannii*		trimethylamine		Chingin et al., 2015
*Streptococcus pneumoniae*		acetaldehyde; ethanol; acetone; dimethyl sulfide		Allardyce et al., 2006
*Neisseria meningitidis*		acetone; dimethyl disulfide		Allardyce et al., 2006

### Gastrointestinal Infections

A diverse number of bacterial pathogens can colonize various parts of the gastrointestinal system causing infections with a wide range of severity (Sweeney and Morton, 2013). The use of fecal volatilome was proposed as a diagnostic approach to differentiate between the gastrointestinal infections and even between infectious and non-infectious diseases.

*Clostridioides difficile* (formerly *Clostridium difficile*) is one of the leading causes of antibiotic-associated diarrhea (Doll et al., 2019). *C. difficile*’s unique fecal volatilome from patients with infectious diarrhea included the furan metabolites, 2-furancarboxaldehyde and 5-methyl-2-furancarboxaldehyde (Probert et al., 2004). Other research groups studied *C. difficile* infections and studied the volatilomes of patients with and without diarrhea. Experiments identified other metabolites and proposed them as markers for *C. difficile* infection. These predictive metabolites for *C. difficile* infection included propan-1-ol, 3-methylbutanal, ethyl propionate, hexanoic acid, *p*-cresol, dodecane, and indole (Patel et al., 2019).

Other diarrhea-causing pathogens (e.g., *Vibrio cholerae* and *Campylobacter jejuni*) have also been studied for their fecal volatilome, however not as heavily as *C. difficile*. For example, the fecal volatilome of *V. cholerae*, which could be confused with other pathogens due to their similar symptoms, was studied. Dimethyl disulfide and *p*-menth-1-en-8-ol were identified as specific markers in this study (Garner et al., 2009). 1-Octen-3-ol was the marker identified for patients infected with *C. jejuni* in comparison to patients with ulcerative colitis or *C. difficile* infection (Garner et al., 2007).

Another interesting application of bacterial volatilome in infection diagnostics is identifying *Helicobacter pylori* infection. *H. pylori* colonizes the stomach causing chronic infections and ulcers (Kusters et al., 2006). By investigating the breath volatilome of infected patients with *H. pylori*, investigators found an elevation in the levels of hydrogen nitrate and hydrogen cyanide (Lechner et al., 2005).

A major challenge with the analysis of fecal volatilome for diagnostics of pathogens in the gut is the absence of a “core” microbiome and “healthy” volatilome that could be used as a basis for differential diagnosis. However, with the advances in the fields of metagenomics and metabolomics, a better understanding of the fecal volatilome and what constitutes a healthy composition is closer to being established. Another challenge in the analysis of fecal volatilome is the high background noise to signal ratio when the targeted volatile is present in minimal concentration, as the fecal volatilome is often dominated by characteristic fermentation volatiles. Lastly, after identifying specific volatiles, investigating their contribution to the virulence of the pathogen in question is another area of research that is worth investigating.

### Respiratory Infections

Respiratory infections are common and recurrent infections in humans and vary greatly in severity from acute to chronic. Due to the similarity of many of their symptoms, differential diagnosis is hard. Breath volatilome is a promising approach to overcome this challenge, especially as it is a non-invasive method. Several efforts have been made to achieve this and identify markers for pathogenic bacteria that infect the respiratory system.

Cystic fibrosis (CF) patients suffer from chronic respiratory infections throughout their lifetime. *Staphylococcus aureus* is one the common pathogens that can infect their lungs during the early stages of the infection (Goss and Muhlebach, 2011). The breath volatilome from CF patients infected with *S. aureus* revealed that undecane and 1,4-pentadiene may serve as markers to identify the culprit in question (Neerincx et al., 2016).

Alongside *S. aureus*, *Pseudomonas aeruginosa* is another major cause of lung infection in CF patients, resulting in high morbidity and mortality rates (Smith et al., 2017). Breath, sputum, or bronchoalveolar lavage fluid from CF patients have been studied to identify any unique *P. aeruginosa* volatilome. Breath volatilome analysis of CF patients infected with *P. aeruginosa* indicated the presence of methyl thiocyanate as a potential marker (Shestivska et al., 2011). Hydrogen cyanide, an infamous volatile has been repeatedly reported in the breath volatilome of patients infected with *P. aeruginosa*, and has been proposed as a non-invasive diagnostic marker (Gilchrist et al., 2013). It is worth noting that hydrogen cyanide was detected in both mouth- and nose-exhaled breath of CF patients including children as well as adults infected with *P. aeruginosa* (Smith et al., 2013). Therefore, this volatile could be used as a diagnostic tool to detect *P. aeruginosa* lung infection in early stages and monitor its eradication. Another interesting marker that has been identified in the breath of CF patients is 2-aminoacetophenone (Scott-Thomas et al., 2010). It is an interesting molecule because of its “grape-like” odor and is known to modulate the virulence of *P. aeruginosa* by shifting it toward a chronic infection phenotype (Kesarwani et al., 2011). Additionally, a few volatile markers have also been identified by analyzing the sputum of CF patients. 2-Hexanone is one of these volatiles that has been identified (Goeminne et al., 2012). Moreover, 2-nonanone is another volatile that was identified as a *P. aeruginosa* emitted volatile from CF patients sputum (Savelev et al., 2011). Finally, examining the bronchoalveolar lavage fluid volatilome from CF patients led to the identification of 2-butanone and 3-methyl-2-butanone as good volatile predictors for *P. aeruginosa* lung infection (Nasir et al., 2018).

*Acinetobacter baumannii* is another multidrug-resistant pathogen. It is often identified as a cause of ventilator-associated pneumonia (Falagas and Rafailidis, 2007). Breath volatilome analysis of patients with *A. baumannii* infection emitted characteristic markers, i.e., 1-undecene, nonanal, decanal, 2,6,10-trimethyl-dodecane, 5-methyl-5-propyl-non-ane, longifolene, tetradecane, and 2-butyl-1-octanol (Gao et al., 2016).

One specific respiratory infection that is a threat to humans is tuberculosis, which is one of the most common causes of death among infectious diseases and is caused by *Mycobacterium tuberculosis* (Khatua et al., 2017). There has been a growing interest in diagnosing this infection using breath volatilome. Numerous volatiles have been proposed as markers for the detection of tuberculosis including naphthalene, 1-methyl-cyclohexane, and 1,4-dimethyl-cyclohexane (Phillips et al., 2007). In another study, methyl phenyl-acetate, methyl nicotinate, methyl *p*-anisate, and *o*-phenylanisole were identified as potential markers for this respiratory disease (Syhre and Chambers, 2008).

Although a considerable number of volatiles have been identified as markers for the presence of certain pathogen, there are numerous challenges associated with the development and use of these markers in the diagnostics of lung infections. These challenges comprise the non-specificity of volatiles, confounding factors, and inconsistencies between *in vitro* and *in vivo* studies. For example, some of the identified volatiles have been shown to be non-specific for the pathogens studied, as in the case of undecane. Undecane, a molecule that was identified as a marker for the presence of *S. aureus* in the breath of infected patients, was also characteristic for lung cancer and chronic obstructive pulmonary disease patients (Phillips et al., 1999; Van Berkel et al., 2010). A similar case of non-specificity was noticed for heptanal, a marker for *M. tuberculosis*, as it also appeared to be marker in patients with lung cancer (Pereira et al., 2015; Mellors et al., 2018). With the failure in identifying one unique volatile to a certain pathogen, a concurrent success has been witnessed in using the whole volatilome fingerprint instead (Kramer et al., 2015). Numerous confounding factors such as diet and smoking have been encountered in the search for specific breath volatilome to specific lung pathogens (Fischer et al., 2015). For example, cigarette smoking induces some of the volatiles that were thought to be specific to *M. tuberculosis*, i.e., methyl nicotinate, methyl salicylate, and methyl phenylacetate, in the breath of smokers (Scott-Thomas et al., 2013). Lastly, a large number of studies that were conducted *in vitro* to identify unique markers to certain pathogens have failed to be translated *in vivo* in the identification of similar volatilomes. Therefore, it is important to consider the environment, including culture conditions, metabolic niches, available nutrients, and host factors when evaluating a pathogen’s volatilome (Dang et al., 2013; Zhu et al., 2013; Gao et al., 2016).

### Bloodstream Infections

Bacterial bloodstream infections, a.k.a. bacteremia, are acute infections that are often lethal and require urgent attention and treatment. Diagnosis of bloodstream infections is heavily based on culturing, which is time consuming and delays appropriate and necessary treatment. The identification of markers that detect such conditions is still a bottleneck in diagnosis. The approach to identify the volatilomes of bacterial bloodstream infections can be simply described as follows: obtain blood from donors, inoculate the blood with the target bacterium, and incubate for a period of time before volatilome analysis.

*Escherichia coli*, a pathogenic Gram-negative bacterium, is responsible for many nosocomial infections and has been used extensively as a model organism to explore new methods for its early detection in bloodstream infections. Incubating *E. coli* in blood from healthy volunteers identified dimethyl sulfide, carbon disulfide, ethanol, acetaldehyde, and methyl butanoate as potential bloodstream infection markers (Umber et al., 2013). Indole is another volatile that has been evaluated using the same model and proposed as a specific marker to *E. coli* (Chingin et al., 2015; Zhong et al., 2019). In another study the following volatiles have been detected at high levels: acetaldehyde, ethanol, acetone, hydrogen sulfide, methanethiol, and dimethyl sulfide (Allardyce et al., 2006). As for *P. aeruginosa*, its blood cultures were characterized by elevated levels of acetic acid and acetone (Allardyce et al., 2006). Moreover, another study detected 1-vinyl aziridine and trimethylamine as specific marker for *P. aeruginosa* in bloodstream infections (Chingin et al., 2015).

Unique volatiles that are associated with *S. aureus* bloodstream infection comprised butyric acid and isovaleric acid (Chingin et al., 2015). Additionally, the volatiles acetaldehyde, ethanol, ammonia, methanethiol, and dimethyl sulfide, were also found to be associated with *S. aureus* (Allardyce et al., 2006).

Furthermore, other pathogenic bacteria were investigated for their specific volatiles when incubated in blood. For *A. baumannii*, trimethylamine was identified (Chingin et al., 2015), while the blood culture for *Streptococcus pneumoniae* resulted in a high level of acetaldehyde, ethanol, acetone, and dimethyl sulfide (Allardyce et al., 2006). As for *Neisseria meningitidis*, high levels of acetone and dimethyl disulfide were observed in blood cultures (Allardyce et al., 2006).

Despite the efforts made in the analysis of blood volatilomes after culturing with different pathogens, a few issues have emerged that are worth discussing. Some of the previously identified mVOCs are non-specific as they were produced by several bacterial pathogens in blood cultures, therefore, it is suggested to consider these volatiles as general markers for bacteremia. Also, the detection of the volatiles in non-infected blood or under metabolic disorder conditions makes the use of these volatiles challenging. Another major limitation is that previously discussed studies used blood samples from healthy volunteers. However, it is known that bloodstream infections occur to immune-compromised patients who also suffer from a wide range of metabolomic changes in their blood, which could be sensed by the pathogen, consequently affecting its metabolism and volatilome (Kruczek et al., 2016; Elmassry et al., 2019, 2020). Therefore, the aforementioned studies are lacking in providing with reliable and specific markers for the detection of bloodstream pathogens.

### Promising Areas of Research in Bacterial Infection Volatilome

Bacterial volatilome analysis has many applications in diagnostics. Some of these applications have been extensively studied and reviewed here while several others are still awaiting investigation, but nevertheless promising. Herein, we will briefly shed light on some of these applications, most of which are based on utilizing the volatilome of body secretions.

Wounds are a major burden on healthcare systems because their treatment is often complicated with chronic infections and potentially the development of biofilms, which makes their eradication arduous. The volatilomes of *S. aureus* and *P. aeruginosa* biofilms have been recently studied for their potential in monitoring biofilm development and response to various treatments (Ashrafi et al., 2018, 2019).

2-aminoacetophenone, a unique volatile to *P. aeruginosa*, was assessed as a marker for *otitis externa*, ear inflammation caused by *P. aeruginosa* infection. This was accomplished using a biosensor for 2-aminoacetophenone that was applied to pus samples from patients. The results were promising for further development of an easy-to-use device that is specific for *P. aeruginosa* ear infection (Kviatkovski et al., 2018).

Skin hygiene, specifically axillary malodor has been an enduring cosmetic concern. This is another promising area of research that requires an interdisciplinary approach to understand, first, the skin microbiome, second, its volatilome. Previous work has investigated the axillary malodor volatilome, which comprised organic acids, ketones, aldehydes, and sulfur-containing compounds (Callewaert et al., 2014). In parallel, researchers have also investigated the axillary microbiome and found a strong correlation between axillary malodor and low abundant species such as *Staphylococcus hominis* (Troccaz et al., 2015). From a more recent study it has been shown that *S. hominis* is able to release the volatile compound 3-methyl-3-sulfanylhexan-1-ol, a contributor to axillary malodor, from its precursor (Minhas et al., 2018).

Oral hygiene is a daily concern to millions of humans. One of the key issues relevant to oral hygiene is malodorous breath. Malodorous breath, a.k.a. bad breath or halitosis, is a frequent oral problem, in which people suffer from an unpleasant breath odor, which often interferes with their social life (Kapoor et al., 2016). Breath and saliva volatilomes have been studied to characterize this problem and its associated oral microbiome, which is another exciting area of research that is still in an early phase of development (Payne et al., 2000). There is evidence linking malodorous breath to certain species in the saliva microbiome (Takeshita et al., 2010). Moreover, recent research was able to connect certain pathogens such as *Porphyromonas gingivalis* and its volatile sulfur compound, 3-bromo-5-chloro-1-tosyl-1H-pyrrolo[2,3-b]pyridine, to malodorous breath (Qiao et al., 2018). Dental caries is a recurrent disease, which is associated with poor oral hygiene. Volatilome analysis of *in vitro* cultures of cariogenic species such as *Streptococcus mutans*, *Lactobacillus salivarius*, and *Propionibacterium acidifaciens* have been investigated and associated with unique volatiles. However, the association of those volatiles with caries using *in vivo* studies are yet to be established (Hertel et al., 2016).

Another potential application of bacterial volatilome analysis would be in identifying the causative agent of urinary tract infections. Urine cultures with *E. coli*, *Proteus vulgaris*, *P. aeruginosa*, *S. aureus*, *Staphylococcus epidermidis*, *Klebsiella pneumoniae*, *Enterococcus faecalis* have been conducted and their volatilomes were investigated. The results were encouraging as researchers were able to discriminate between different pathogens based on their volatilome (Storer et al., 2011).

Bacterial vaginosis is a common vaginal infection characterized by fishy odor, thin milky discharge, and irritation, which is caused by *Gardnerella vaginalis* (Bagnall and Rizzolo, 2017). Surprisingly, no studies have been conducted to investigate the volatilome of this pathogen, its disease, or even other pathogens of the urogenital tract such as *Neisseria gonorrhoeae*. Therefore, research in the diagnostics of bacterial vaginosis based on vaginal volatilome analysis will be an interesting area to explore.

Recent literature suggests the involvement of pathogens in neurodegenerative diseases pathology (e.g., Alzheimer’s and Parkinson’s disease) (Quigley, 2017; Dominy et al., 2019; Trivedi et al., 2019). Seborrheic dermatitis, a condition developed by the overproduction of sebum, is a non-motor symptom of Parkinson’s disease (Ravn et al., 2017). Seborrheic dermatitis in Parkinson’s disease patients is associated with a higher abundance of *Malassezia* in their skin microbiome (Ravn et al., 2017). The presence of *Malassezia* was recently correlated with a higher lipase activity and an increase in eicosane (Trivedi et al., 2019). Eicosane was hypothesized to be produced by *Streptomyces* as a defensive mechanism against *Malassezia* due to its antifungal activity (Wikoff et al., 2009; Trivedi et al., 2019). Therefore, it was proposed that eicosane could serve as one of the sebum biomarkers associated with Parkinson’s disease (Trivedi et al., 2019). *P. gingivalis*, a key pathogen in periodontitis, is a key contributor to Alzheimer’s disease pathogenesis (Dominy et al., 2019). Till today, several markers (e.g., methanethiol and acetone) in breath have been proposed for the diagnostic identification of *P. gingivalis* in the oral cavity (Roslund et al., 2019). However, these markers have not been investigated for their association with Alzheimer’s disease. Future studies focusing on the role of these markers in association with Alzheimer’s disease will be significant. The capacity of the gut microbiota to produce volatile short-chain fatty acids like butyrate, received significant attention over the years due to their key role in maintaining healthy physiology (Ríos-Covián et al., 2016). Butyrate is associated with improved cognitive functions and has beneficial effect on brain disorders (Govindarajan et al., 2011; Bourassa et al., 2016). Furthermore, butyrate-producing microbiota were diminished in elder patients with Alzheimer’s disease (Haran et al., 2019). Recently, the butyrate-producing probiotic, *Clostridium butyricum*, was found to have an anti-neuroinflammatory effect in Alzheimer’s disease (Sun et al., 2020). This effect was mediated via butyrate by regulating the gut-brain axis (Sun et al., 2020). Therefore, butyrate and other volatile short-chain fatty acids are promising preventive and therapeutic interventions in Alzheimer’s disease. Overall, these studies provide a critical insight into the interplay between neurodegenerative diseases and the human microbiome. The production of microbial VOCs could affect the disease pathology or be altered in a significant way by the disease itself. This research area has promising major applications in developing novel therapeutic targets or biomarkers.

Herein, we have covered numerous applications for bacterial volatilome analysis in the context of human infections and the advantage of its use in diagnostics. We discussed how some areas of research have quickly advanced, while others are still lacking. Furthermore, we have emphasized the importance of this approach, which after further development could allow for the differential diagnosis of bacterial infections using a rapid, sensitive, and specific point-of-care method.

## Author Contributions

ME and BP contributed the same amount of work to this review.

## Conflict of Interest

The authors declare that the research was conducted in the absence of any commercial or financial relationships that could be construed as a potential conflict of interest.
